# Frequency of Hereditary Hemochromatosis (*HFE*) Gene Mutations in Egyptian Beta Thalassemia Patients and its Relation to Iron Overload

**DOI:** 10.3889/oamjms.2016.055

**Published:** 2016-06-01

**Authors:** Azza Aboul Enein, Nermine A. El Dessouky, Khalda S. Mohamed, Shahira K.A. Botros, Mona F. Abd El Gawad, Mona Hamdy, Nehal Dyaa

**Affiliations:** 1 *Clinical Pathology Department, Kasr El Ainy Medical School, Cairo, Egypt*; 2 *National Research Center, Cairo, Egypt*; 3 *Pediatrics Department, Aboul Riche Pediatric Teaching Hospital, Cairo University, Cairo, Egypt*; 4 *Aboul Riche Pediatric Teaching Hospital, Cairo University, Cairo, Egypt*

**Keywords:** Thalassemia, Iron overload, HFE, genes, PCR

## Abstract

**AIM:**

This study aimed to detect the most common HFE gene mutations (*C282Y*, *H63D*, and *S56C*) in Egyptian beta thalassemia major patients and its relation to their iron status.

**SUBJECTS AND METHODS:**

The study included 50 beta thalassemia major patients and 30 age and sex matched healthy persons as a control group. Serum ferritin, serum iron and TIBC level were measured. Detection of the three *HFE* gene mutations (*C282Y*, *H63D* and *S65C*) was done by PCR-RFLP analysis. Confirmation of positive cases for the mutations was done by sequencing.

**RESULTS:**

Neither homozygote nor carrier status for the *C282Y* or *S65C* alleles was found. The *H63D* heterozygous state was detected in 5/50 (10%) thalassemic patients and in 1/30 (3.3%) controls with no statistically significant difference between patients and control groups (p = 0.22). Significantly higher levels of the serum ferritin and serum iron in patients with this mutation (p = 001).

**CONCLUSION:**

Our results suggest that there is an association between *H63D* mutation and the severity of iron overload in thalassemic patients.

## Introduction

Thalassemias are a group of inherited disorders resulting from reduced rate of globin synthesis. This insufficiency prompts imbalanced globin chain synthesis, decreased the production of hemoglobin and destruction of the red cells or their precursors from the impacts of the globin subunits which are delivered in relative abundance (Fawdry, 1944 [1]).

The clinical picture of β-thalassemia is caused by anemia, blood transfusion and increased body iron. Transfusion therapy has fundamentally expanded the survival of patients with β thalassemia. However, both repeated transfusions and increased gut iron absorption have led to iron overload and an increment in the complications caused by increased total body iron (Borgna-Pignatti *et al.*, 2004 [2]).

Hereditary hemochromatosis (HH) is a genetic disorder characterized by iron overload resulting in multiorgan damage. The gene underlying this disorder has been identified as the *HFE* gene. The *HFE* gene has an important role in iron homeostasis by regulating the absorption of iron. *HFE* gene mutations are currently known as a cause of increased iron absorption, iron overload and HH (Dhillon *et al.*, 2012 [3]).

Among the most frequently encountered mutations in HH are three missense mutations (SNPs) in *HFE* gene with an autosomal recessive pattern. The most common is a mutation within exon 4 of the *HFE* gene, resulting in an amino acid change at position 282 from cysteine to tyrosine (*C282Y*), this mutation was found to be responsible for 60% of HH cases in Mediterranean populations. Another mutation is *H63D* which results in a C-G transition at nucleotide 187 of exon 2 of the *HFE* gene leading to a histidine to aspartic acid substitution.

Iron overload is most pronounced when this allele is combined with *C282Y* allele (*C282Y/H63D*).

The third *HFE* gene mutation is a 193A→T substitution in exon 2 with a resulting serine to cysteine substitution in amino acid position 65 (*S65C*) (Oliveira *et al.*, 2006 [4]).

Many studies have shown that beta-thalassemia patients having *HFE* mutations are more likely to develop iron overload that may necessitate early iron chelation even in the heterozygous state (Sharma et al.,2005 [5]). Different methods can be used to genotype The *HFE* gene; these include RFLP (restriction fragment length polymorphism), DNA sequencing and the improved real-time PCR assay (Moyséset al., 2008 [6]).

This study aimed to detect the most common *HFE* gene mutations (*C282Y, H63D*, and *S56C*) in Egyptian beta- thalassemia major patients and their relation to patients iron status.

## Subjects and Methods

Patients with β thalassemia major (26 males and 24 females) and 30 age and sex matched controls were included in this study. The selected pediatric patients were referred from the Hematology Outpatient Clinic, Abou El Riche New Children Hospital, Cairo University to Human Genetics Department in the National Research Center, Cairo, Egypt.

This study has been approved by the clinical pathology division Cairo University ethics committee and has been performed according to the ethical standards set down in the 1964 Declaration of Helsinki and its later corrections.

Blood samples (10 ml) were collected from patients and controls. Screening for hemoglobinopathies was carried out by hemoglobin electrophoresis (Helena Laboratories, Beaumont, TX, USA), ferritin level assay was carried out by EIA (enzyme immunoassay), and iron and TIBC were assessed by automated analyzer (Olympus AU400). Hematologic values and red cell indices were obtained (ADVIA 120 Hematology System).

As for transferrin saturation, it was calculated based on the equation: serum iron/TIBC × 100.

### Molecular studies

Genomic DNA was extracted from peripheral leukocytes by the phenol-chloroform method. The Primer sequences, PCR product lengths, restriction endonucleases and restriction patterns for the *H63D* and *S65C* and *C282Y* genetic variants are shown in Table 1.

**Table 1:** Primer sequences, PCR product lengths, restriction endonucleases and restriction patterns for the analyzed genetic variants

Mutation	Forward primer	Reverse primer	Product length (bp)	Restriction enzyme	Digested fragments length (bp)
*H63D*	5’ACATGGTTAAGGCCTGTTGC3’	5’GCCACATCTGGCTTGAAATT3’	208	BclI	Not cut
*S65C*	5’ACATGGTTAAGGCCTGTTGC3	5’GCCACATCTGGCTTGAAATT3	208	HinfI	Not cut
*C282Y*	5’TGGCAAGGGTAAACAGATCC3’	5’CTCAGGCACTCCTCTCAACC3’	400	SnabI	110, 290 and 400

**Figure 1:**
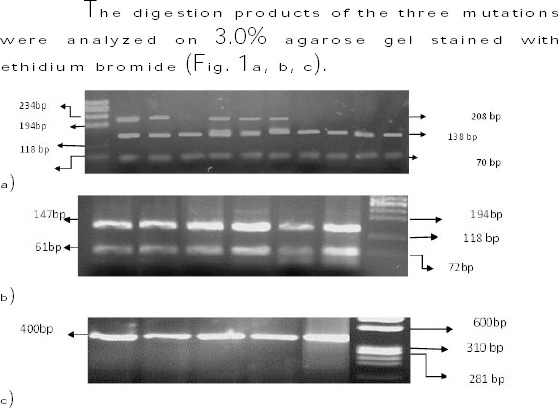
*The digestion products of the three mutations were analyzed on 3.0% agarose gel stained with ethidium bromide*.

The digestion products of the three mutations were analyzed on 3.0% agarose gel stained with ethidium bromide (Fig. 1a, b, c).

### DNA sequencing

DNA Sequencing was done for confirmation of the positive cases for mutations. Purification of PCR products to remove dNTPs and primers was performed using QIAquick PCR purification kit (Cat no. 28104). Cycle sequence PCR was carried out using Big Dye R Terminator kit (Cat no. 4336917-USA). Cycle sequencing PCR was carried out on Perkin-Elmer thermal cycler (Applied Biosystem 2720, Singapore) using the following condition: Denaturation at 96°C for 1 min followed by 25 cycles of denaturation at 96°C for 10 sec, annealing at 56°C for 5 sec, and elongation at 60°C for 4 min. The samples were injected into automatic sequencer and data were analyzed using ABI Prism DNA sequencing analysis software.

**Figure 2:**
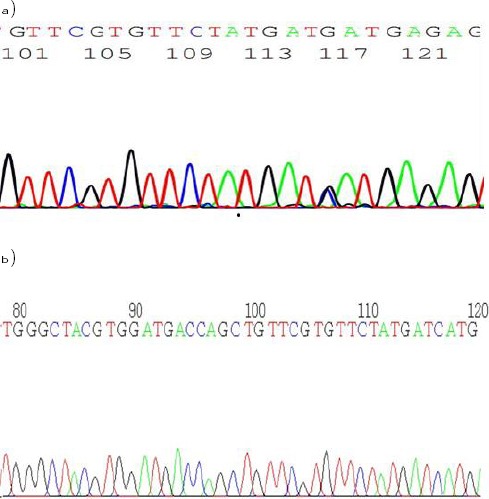
*The presence of overlapping G and C peaks indicate normal and mutant sequence of H63D mutation (a) whereas negative cases showed only C peak (b)*.

As regards the H63D mutation, the presence of overlapping G and C peaks indicate the normal and mutant sequence of H63D mutation (Fig. 2a) whereas negative cases showed only C peak (Fig. 2b).

### Statistical analysis

Data was analyzed using IBM advanced SPSS statistical package version 20 (SPSS Inc., Chicago, IL). Numerical data were expressed as a mean and standard deviation. Qualitative data were expressed as frequency and percentage.

Data were analyzed by the chi-square test for multiple independent samples to identify the relationship between the mutations and the groups studied. Fisher’s exact test was used to compare results between the two groups. A P value < 0.05 was considered significant

## Results

The clinical and laboratory data of the studied patients are shown in Table 2

**Table 2:** Clinical and laboratory data of the studied patients

	Minimum	Maximum	Mean ± Std deviation
Age (year)	11	40	27.2 ± 7
Age at onset (month)	2	36	11.1 ± 9.5
Frequency of blood transfusion/year	8	24	14.8 ± 6.3
Hb level (g/dl)	5.6	10	7.8 ± 1.2
Ferritin (ng/ml)	723	7655	3487.3 ± 1886.1
Serum iron (ug/dl)	50	348	205.3 ± 71.3
TIBC (ng/ml)	180	400	273 ± 59.4
Transferrin saturation%	20	106.6	75.1 ± 17.4

Normal s. iron: - males 65-175 μg/dL; Females 50-170 μg/dL, TIBC: 20 to 150 ng/mL; ferritin: male: 12-300 ng/mL; female: 12-150 ng/mL, transferrin saturation: 20–50%.

In the current study, all thalassemia major patients had elevated serum ferritin level, (10%) of patients had serum ferritin level < 1000 ng/ml, (30%) had serum ferritin between 1000 to 2500 ng/ml, while (60%) had values above 2500 ng/ml.

Transferrin saturation was above 50% in 48/50 (96%), only two cases (4%) had transferrin saturation below 50%.

### Genotyping of S65C and C282Y mutation

The *S65C* and *C282Y* mutations were absent in all beta thalassemia major patients as well as in the control group.

### Genotyping of H63D mutation

Allelic frequency of *H63D* mutation was found to be 10%, (5/50) in beta thalassemia major patients, heterozygous HD genotype, while the rest 90%, (45/50), showed no mutation HH genotype. None of the patients showed the HH homotype.

As for control group only 1 individual (1/30), 3.3% showed the heterotype HD for *H63D* genotyping.

Analysis of genotypes of this mutation revealed that the frequency of HD genotype was higher among beta-thalassemia major patients (5 of 50), 10% than healthy controls (1 of 30), 3.3%. There was no statistically significant difference in the frequency of HH, HD or DD genotypes between thalassemia patients and the control group (Table 3).

**Table 3:** Distribution of *H63D* genotype and alleles between thalassemic patients and controls

	Thalassemic patients (N = 50)		Controls (N = 30)		P value
*H63D* genotype	Number	Frequency	Number	Frequency	
H/H	45	90%	29	96.70%	
H/D	5	10%	1	3.30%	0.22
D/D	0	0	0	0	

*H63D* alleles	Number	Frequency	Number	Frequency	
H	95	95%	59	97.30%	
D	5	5%	1	1.70%	0.08

There was a statistically significant difference on comparing beta-thalassemia patients with wild HH genotype and those carrying the heterozygous mutation HD genotype as regard the serum iron and serum ferritin levels which were significantly higher in HD genotype (Table 4).

**Table 4:** Comparison between hematological and biochemical measurements in different *H63D* genotypes in beta-thalassemia patients

Parameter	H/H mean ± SD N = 45	H/D mean ± SD N = 5	P value
Ferritin (ng/ml)	3121.8 ± 1600	6778 ± 581	0.01**

Serum iron (ug/dL)	194.6 ± 66.2	319.2 ± 58.2	0.01**

TIBC (ng/ml)	265.4 ± 56.8	296.8 ± 55.3	0.07

Transferrin saturation (%)	73.5 ± 17	83.2 ± 7.1	0.23

* p < 0.05 = significant.

## Discussion

Iron overload is the main cause of significant morbidity and mortality in thalassemia. The progressive iron overload in beta-thalassemia major patients results from ineffective erythropoiesis increased absorption of iron by the gastrointestinal tract, defective physiologic excretion of excess iron, and essentially, multiple blood transfusions (Giardina and Grady 2008 [7]).

Hereditary hemochromatosis is an autosomal recessive disease characterized by increased intestinal iron absorption and progressive iron overload (Jowkar et al., 2011 [8]). Mutations in the *HFE* gene, known to be significantly associated with hereditary hemochromatosis, were found to have a modifying effect on iron absorption in thalassemia patients (Camaschella et al., 2002 [9]).

In this study, we have investigated the possible association between iron overload and three mutations in *HFE* gene (*C282Y, H63D* and *S65C* mutations) in beta thalassemia major patients.

We found that all thalassemia major patients had elevated serum ferritin, where 5/50 (10%) of patients had serum ferritin level < 1000 ng/ml, 15/50 (30%) had serum ferritin between 1000 to 2500 ng/ml, while 30/50 (60%) had values above 2500 ng/ml.

The serum iron and TIBC levels in the present study were 205.3 ± 71.3 mg/dl and 273 ± 59.4 mg/dl respectively, iron chelation therapy was not equally efficacious in the cases as reflected by variable serum iron, TIBC, and serum ferritin. This could be explained by the fact that the clinical phenotype of homozygous beta-thalassemia may be altered when other genetic variants present outside the globin clusters are co-inherited as well. These genetic modifiers influence mainly impact for the most part of the thalassemia complications (Galanello and Origa 2010 [10]).

In this study, the three mutations in *HFE* gene (*C282Y, H63D* and *S65C* mutations) were screened in 50 patients and our results have been compared with 30 healthy individuals which were taken as a control group.

The screening of *C282Y* and *S65C* mutations revealed that these mutations were not found in the thalassemia or control group.

Our finding is concurrent with that of Karimi et al, (2004) who studied 203 normal adults and 154 transfused patients affected with β-thalassemia in Iran and the two mutations were not present in both groups [11].

Also in agreement with our study, the *C282Y* mutation was not observed in another study done in Tunis on 50 beta thalassemia patients (Mellouli et al., 2006 [12]).

In another study done by Kaur et al, (2004), who studied Asian-African and Middle Eastern origins living in the USA, it was found that 2 out of 81 patients were positive for *C282Y* mutation while *H63D* mutation was not observed (Kaur and Andersen 2004) [13]. This finding confirms the rarity of *C282Y* mutation in the African populations.

In the current study, *H63D* mutation was found in 5/50 thalassemia patients (10%) compared to 1/30 in the control group (3.3%) with allele frequency 0.5% in beta-thalassemia patients and (1.7%) in the control group.

Another study in India revealed a higher frequency of *H63D* mutation, they studied 75 thalassemia major patients in North India and reported that H63D mutation was observed in 12% patients compared to 8.6% in a control group of India [14].

In agreement with our results the allele frequencies in thalassemia patients in Brazil were 0.29%, 13.70% and 0.60% for the *C282Y, H63D* and *S65C* mutation respectively [15].

In the present study, there was no statistically significant difference in the frequency of *H63D* mutation between β-thalassemic patients and the control group (p = 0.22).

In close agreement with our results, previous studies in Tunis and India reported that there was no significant difference in the prevalence of HH, HD or DD genotypes between thalassemia patients and healthy controls (Kaur D and Andersen. 2004 [13]).

However, the role of *HFE* gene mutations on developing iron overload was previously studied in 41 beta-thalassaemia carriers in Egypt and it was found to be more common among beta-thalassaemia carriers compared with normal controls (*H63D, S65C*, and *C282Y* allele frequencies were 30.5%, 13.4% and 7.3% respectively in beta-thalassaemia carriers and 10.0%, 2.5% and 0.0% respectively in the control group). This difference may be related to the limited sample size in both studies, in addition, the verification of the presence of mutation by sequencing in our study is more in favor for a lower frequency rate (Madani et al.,2011 [15]).

Interestingly, there was a significant difference between serum ferritin level and serum iron in beta-thalassemia patients with and without the *H63D* mutation (P = 0.001). We found that in the group with *H63D* mutation patients had serum ferritin above 2500 ng/ml (p < 0.05).

These findings point to a possible association between *H63D* mutation and the severity of iron overload in thalassemic patients.

A similar result was also described by Melis et al, (2002) [14], who suggested that the *H63D* mutation may have a modifying effect on iron absorption and that the inheritance of *H63D* mutation may aggravate the beta-thalassemia trait even if present in heterozygous state (Melis et al., 2002) [14].

Also in agreement with our results Sharma et al, (2007) concluded that thalassemia intermedia patients with co-existent *HFE* mutation are more likely to develop iron overload and may need early iron chelation therapy [5].

A different finding was observed in previous studies by Mellouli et al, (2006), who suggested that the inheritance of *H63D* mutation does not influence the severity of iron overload in beta-thalassemia patients (Mellouli et al., 2006) [12].

Also, Garewal et al, (2005) suggested that the presence of *H63D* mutation does not increase body iron as measured by serum ferritin in beta thalassemia [16].

Previous studies have yielded clashing results: some proposed iron overload may emerge from the association of the β-thalassemia with homozygosity or even heterozygosity for *HFE* mutations and others claimed that no relation exists between total body iron and *HFE* genotypes (López-Escribano et al., 2012 [17])

The discrepancy between our results and the previous results has several explanations as the expression of *HFE* hemochromatosis is modified by genetic and acquired factors, for example, dietary habits, blood loss or donation, pregnancy, menopause, malabsorption (Sharma et al.,2007 [5]).

As a second explanation, mutations in the *HFE* gene other than those tested in this study may be responsible for the iron overload and to higher serum ferritin.

Our study suggests that somehow, *HFE* gene mutations could be involved in increasing iron storage possibly through interaction with other genetic determinants of β-thalassaemia. This can lead to iron overload with secondary tissue harm in multiple body organs creating most of morbidity and mortality in thalassemia patients.

Therefore, this study supports the importance of screening of *HFE* gene mutations in thalassemia patients for detection of those having a higher likelihood of developing iron overload even when the mutation is present in the heterozygous state.

This may enhance the quality of life in these patients by frequent follow-up for prior detection of iron overload and its complications and by applying more aggressive iron chelating protocols.

Recommendations: This study should be expanded to include a larger number of patients as well as a control population. We recommend testing of patients with thalassemia “intermedia and minor” for the presence of *HFE* gene mutations and correlate its presence with the serum ferritin level.

Also, we need testing for other *HFE* gene mutations or other genes involved in hereditary hemochromatosis in the iron-overloaded patients, who were negative for the *C282Y, H63D* and *S65C* mutations.
